# Effect of the psychosocial adjustment program on midwifery students’ anxiety and psychological well-being during the COVID-19 pandemic

**DOI:** 10.18332/ejm/146735

**Published:** 2022-06-07

**Authors:** Emel Bahadır-Yılmaz, Arzu Yüksel

**Affiliations:** 1 Department of Psychiatric Nursing, Faculty of Health Sciences, Giresun University, Giresun, Turkey; 2 Department of Psychiatric Nursing, Faculty of Health Sciences, Aksaray University, Aksaray, Turkey

**Keywords:** psychosocial adjustment, trait anxiety, psychological well-being, state anxiety, midwifery students

**Dear Editor,**

During the COVID-19 pandemic, midwifery students experienced some problems, including moving to online education, being isolated from peers, and having household responsibilities and financial concerns^1^. They reported difficulty concentrating and learning, fear of infection, and worries about their family well-being^2^. They also experienced practical worries, fewer clinical learning opportunities, and fundamental doubts about their choice to become a professional^3^. Owing to distance education, students are less exposed to clinical practice and face internet limitations, economic difficulties, and mental problems such as anxiety and stress^4^.

This study aimed to assess the effect of the psychosocial adjustment program (PSAP) on midwifery students’ anxiety and psychological well-being. This study is a single-group experimental study with a pre-test and post-test. The study was conducted during the 2020–2021 academic year. The study population consisted of 91 first-year midwifery students enrolled in the Faculty of Health Sciences at a state university in northeastern Turkey. Sixteen-seven first-year midwifery students volunteered to participate in the study. Students completed three questionnaires, namely, the Student Information Form, State–Trait Anxiety Inventory^5,6^, and Psychological Well-being Scale^7,8^.

Data were collected from the students through an online survey, for which questionnaires were created using Google Forms between October 2020 and February 2021. The Psychosocial Adjustment Program consisted of eight weekly sessions (Table 1) and was conducted on Google Meet. Data were collected online. All students were informed about the purpose of the study through Google Forms. Data were analyzed using the SPSS 24 package program. Descriptive statistics, Kolmogorov–Smirnov test, and Wilcoxon’s signed-ranks test were used in data analysis.

**Table 1:** The sessions and contents of the psychosocial adjustment program

*Session*	*Content*
1	Introducing the students, sharing reasons for choosing the department, and sharing concerns about midwifery education.
2	Introducing the department and department lecturers, giving information about the courses, and providing suggestions about midwifery education by lecturers.
3	Inviting a graduate midwife student, sharing her experiences and suggestions, and answering students’ questions.
4	Watching videos on time management, discussing time management, and making suggestions about time management.
5	Discussing motivation, making a work plan, and setting short- and long-term goals regarding the training process and for the future.
6	Dealing with educational stress, sharing experiences with stress management, and talking about effective coping methods.
7	Discussing resilience, sharing the life stories of resilient people, and discovering our strengths and achievements.
8	Doing occupational therapy and talking about its benefits, evaluating the program, and getting feedback from the students.

All students were female, and the mean age of participants was 19.03 ± 1.62 years. The mean State Anxiety sub-dimension score at the beginning of the program was 41.00 ± 10.37, and that at the end of the program was 35.88 ± 8.38. The difference between pretest and posttest score averages was significant (z= −4.140, p<0.001). The mean Trait Anxiety sub-dimension scores of the students were 44.32 ± 4.58 and 41.94 ± 3.41 at the beginning and end of the program, respectively. The difference between mean pretest and posttest scores was significant (z= −3.522, p<0.001). The mean Psychological Well-being Scale posttest score (46.38 ± 4.87) was higher than the mean pretest score (44.76 ± 6.95), and the difference between the mean pretest and posttest scores was significant (z= −2.718, p=0.007).

In this study, the Psychosocial Adjustment Program decreased midwifery students’ state and trait anxiety and increased their psychological well-being. This study also provided a web-based intervention model during the COVID-19 pandemic, decreasing anxiety levels, and increasing psychological well-being. For this reason, The Psychosocial Adjustment Program can be used and developed to reduce the state anxiety and trait anxiety and improve the psychological well-being of midwifery students.

## Data Availability

The data supporting this research are available from the authors on reasonable request.
